# Patients with colorectal cancer combined with HIV had a worse overall survival after surgery: a meta-analysis

**DOI:** 10.3389/fonc.2025.1440105

**Published:** 2025-01-29

**Authors:** Wen-Wen Yang, Xiong Zhou, Gan He

**Affiliations:** ^1^ Department of Ophthalmology, Chongqing University Center Hospital, Chongqing Emergency Medical Center, Chongqing Fourth People’s Hospital, Chongqing, China; ^2^ Department of Hepatobiliary Surgery, The Second Affiliated Hospital of Chongqing Medical University, Chongqing, China; ^3^ Department of Gastrointestinal Surgery, Yongchuan Hospital of Chongqing Medical University, Chongqing, China

**Keywords:** HIV, colorectal cancer, surgery, overall survival, infection

## Abstract

**Purpose:**

The purpose of this current study was to find out whether human immunodeficiency virus (HIV) affected overall survival (OS) of colorectal cancer (CRC) patients after surgery.

**Methods:**

PubMed, Embase, the Cochrane Library, and CNKI were searched from inception to March 27, 2023 to find eligible studies. Eligible studies included CRC patients grouped by HIV status (HIV-positive and HIV-negative). Stata SE 16 was used for data analysis.

**Results:**

A total of eight studies involving 2180 patients were enrolled in this study. After data analysis, there were significant differences in sex (OR=0.69, 95% CI=0.49 to 0.98, I^2^ = 22.6%, P=0.04<0.1), tumor grade (OR=6.61, 95% CI=2.36 to 18.49, I^2^ = 0.00%, P=0.00<0.1), and tumor location (OR=2.19, 95% CI=1.74 to 2.77, I^2^ = 0.04%, P=0.00<0.1) between the HIV and non-HIV groups. Furthermore, we found that HIV was associated with worse OS in CRC patients after surgery (HR=3.12, 95% CI=2.07 to 4.69, I^2^ = 52.51%, P=0.00<0.1).

**Conclusion:**

This study highlights that HIV is associated with significantly poorer OS in CRC patients after surgery, emphasizing the need for tailored postoperative management strategies for this vulnerable population. Future research should explore underlying mechanisms and potential interventions to improve outcomes for HIV-positive CRC patients.

## 1 Introduction

In recent years, with the use of highly active antiretroviral therapy (HAART), the survival of human immunodeficiency virus (HIV) -infected patients has been significantly increased (1–4). This has led to an increased chance of malignancy in HIV-infected people as well, especially in non-HIV related malignancy (5, 6).

Colorectal cancer (CRC) is one of the most common malignancies in the world and the second leading cause of cancer-related deaths (7, 8). Currently, there are approximately 185 million CRC patients worldwide (9). Surgical resection remains the cornerstone of colorectal cancer treatment (10, 11). Previous studies have shown that the incidence of CRC is higher in people with HIV compared to the general population (12–14). As the life expectancy of HIV patients increases, it is important to understand the epidemiology and natural course of CRC in HIV-infected patients and its treatment.

The impact of HIV on postoperative overall survival (OS) in CRC patients remained controversial in previous studies. Berretta M et al. (15) and Sigel C et al. (16) found that OS after surgery was worse in HIV-infected CRC patients than in general CRC patients. Other studies reported there was no significant difference of HIV on OS in CRC patients after surgery (17–21). Therefore, this study aimed to determine whether HIV affects the overall survival of CRC patients post-surgery and to identify factors contributing to worse outcomes.

## 2 Methods

This study was performed with the Preferred Reporting Items for Systematic Reviews and Meta-Analyses (PRISMA) statement (22).

### 2.1 Search strategy

Four databases including PubMed, Embase, the Cochrane Library, and CNKI were searched from inception to March 27, 2023. Two key words were as follows: HIV and CRC. As for HIV, the search strategy was as follows: (HIV OR human immunodeficiency virus OR AIDS OR acquired immunodeficiency syndrome); in terms of CRC, we searched: (colorectal cancer OR colorectal neoplasm OR colorectal tumor OR colorectal carcinoma). Finally, we used “AND” to connect the key words. Titles and abstracts were limited. English and Chinese were the limited languages.

### 2.2 Inclusion and exclusion criteria

Inclusion and exclusion criteria were used to screen potential articles. The inclusion criteria were as follows: 1, all patients were diagnosed with CRC and underwent surgery; and 2, both the HIV group and the non-HIV group were reported. The exclusion criteria were as follows: 1, case reports, case series, comments, letters to the editor, conference abstracts and nonoriginal articles; 2, data were repeated or overlapped; and 3, incomplete data.

### 2.3 Study selection

Four databases were independently searched and screened for eligible studies by two researchers. First, duplicate studies were excluded. Then, the two researchers scanned the titles and abstracts to exclude ineligible studies according to the inclusion and exclusion criteria. Finally, full text would be read to identify studies that could be included. The group discussion resolved all differences.

### 2.4 Data collection

The information contained baseline characteristics of the included studies and patients. The studies’ characteristics included first author, published year, country, study date, study type, sample size, language of the studies, and Newcastle-Ottawa Scale (NOS) score. As for patients’ baseline information, age, sex, tumor stage, grade, and location were collected. As for prognosis, we included OS.

### 2.5 Quality assessment

NOS was used to assess the quality of included studies (23). Classification of eligible articles as high quality (9 points), medium quality (7-8 points), or low quality (less than 7 points) based on NOS score.

### 2.6 Statistical analysis

Dichotomous variables were described by odds ratios (ORs) and 95% confidence intervals (CIs). Mean differences (MDs) and 95% CIs were calculated for continuous variables. Hazard ratios (HRs) and 95% CIs were used to calculate prognosis of patients. To evaluate the statistical heterogeneity, the I^2^ value and the chi-squared test were used (24, 25). We used the random effects model only to deliver more conservative conclusions, and p<0.1 was considered statistically significant. Stata SE 16 was used for data analysis.

## 3 Results

### 3.1 Study selection

**Figure 1:**
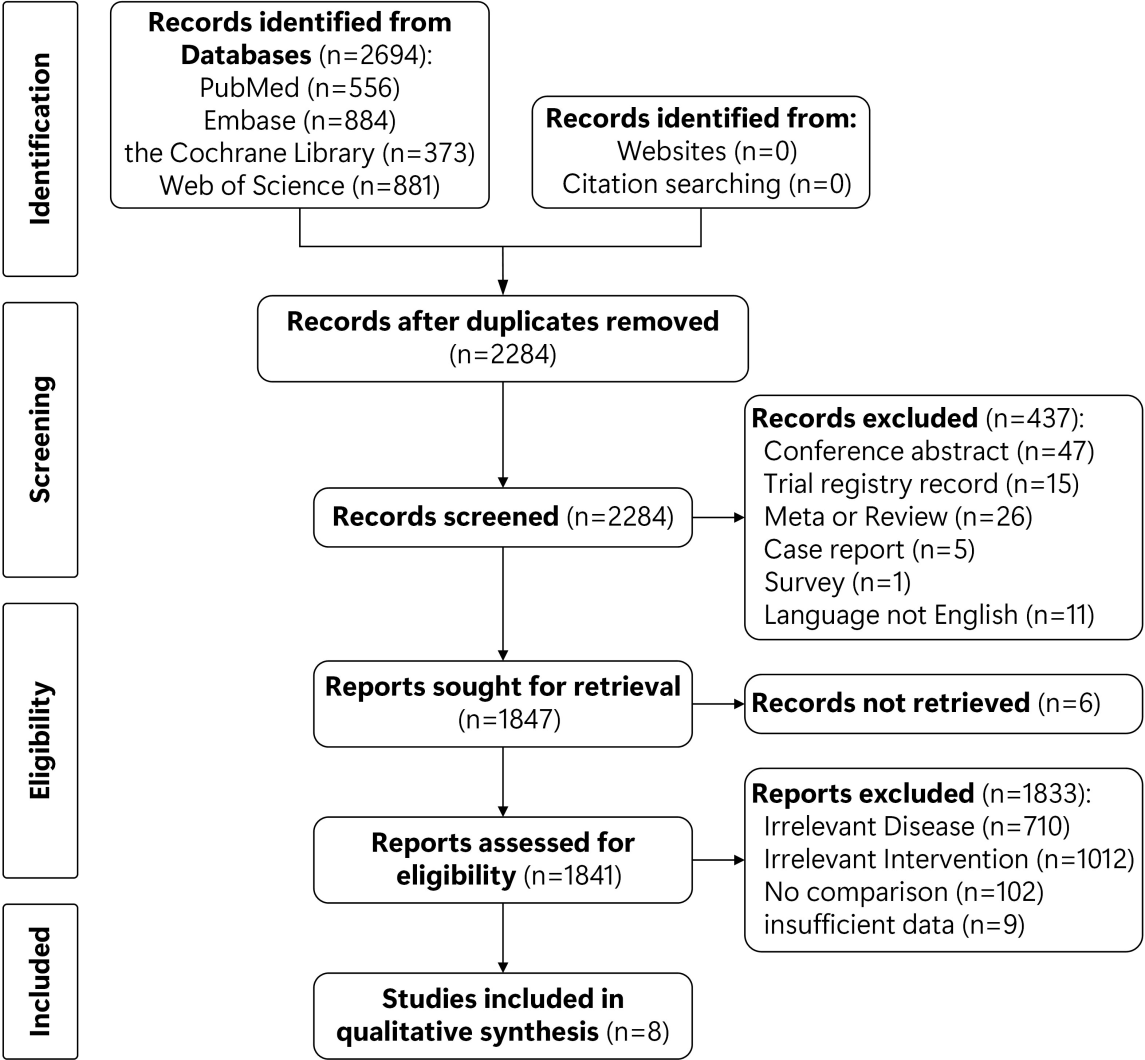
Flowchart of study selection.

A total of 2694 studies were searched from four databases (556 in PubMed, 884 in Embase, 373 in the Cochrane Library, 881 in CNKI) on March 27, 2023. 2284 records were left after duplicates removed. Then, 437 unqualified studies were excluded. After screening the titles and abstracts, 17 studies were left for full text accessed. Finally, eight eligible studies were enrolled in this study (15–21, 26) (**Figure 1**).

### 3.2 Baseline characteristics of the included studies

There were eight studies involving 2180 patients enrolled, 493 patients in the HIV group and 1687 patients in the non-HIV group. These studies were firstly published in 2007 and most recently in 2022. The studies were published in Israel, the United States America (USA), Japan, South Africa, China, and some other Europe countries. The study date was from 1985 to 2019. All the studies were retrospective studies. More details (sample size, language of studies and NOS score) were shown in **Table 1**.

**Table 1:** Baseline characteristics of included studies.

Author	Year	Country	Study date	Study type	Sample size		Language	NOS
					HIV	Non-HIV		
Wasserberg N	2007	Israel	1994-2003	retrospective	11	22	English	9
Berretta M	2009	Europe	1985-2003	retrospective	27	54	English	9
Kumar A	2012	USA	2000-2009	retrospective	17	42	English	8
Hamada Y	2014	Japan	2009-2012	retrospective	177	177	English	9
Sigel C	2016	USA	1993-2014	retrospective	38	146	English	7
Pillay SK	2022	South Africa	2000-2019	retrospective	145	570	English	9
Marcus JL	2016	USA	1996-2011	retrospective	53	646	English	8
Jian-Ning Z	2018	China	2012-2015	retrospective	25	30	Chinese	7

HIV, human immunodeficiency virus; USA, the United States of America; NOS, Newcastle-Ottawa Scales.

### 3.3 Baseline characteristics of the included patients

In terms of the patients’ baseline characteristics, we pooled up age, sex, tumor stage, tumor grade, and tumor location. After data analysis, there were significant differences in sex (OR=0.69, 95% CI=0.49 to 0.98, I^2^ = 22.6%, P=0.04<0.1), tumor grade (OR=6.61, 95% CI=2.36 to 2.24, I^2^ = 0.00%, P=0.00<0.1), and tumor location (OR=2.19, 95% CI=1.74 to 2.77, I^2^ = 0.04%, P=0.00<0.1) between the HIV and non-HIV groups. Age (MD=-0.49, 95% CI=-1.43 to 0.69, I^2^ = 98.33%, P=0.49>0.1) and tumor stage (OR=1.23, 95% CI=0.68 to 2.24, I^2^ = 51.57%, P=0.49>0.1) were not statistically different between the two groups (**Table 2**).

**Table 2:** Baseline information of the patients in the HIV and non-HIV groups.

Characteristics	Studies	Participants (HIV/Non-HIV)	Odds Ratio/Mean Difference (95% CI)	Heterogeneity
Age	6	423/999	-0.49 [-1.43, 0.69]; P=0.49	I^2^ = 98.33%; P=0.00
Sex (male)	8	493/1687	0.69 [0.49, 0.98]; P=0.04^*^	I^2^ = 22.60%; P=0.25
Tumor stage (≥3)	4	NA	1.23 [0.68, 2.24]; P=0.49	I^2^ = 51.57%; P=0.10
Tumor grade (≥3)	2	NA	6.61 [2.36, 18.49]; P=0.00^*^	I^2^ = 0.00%; P=0.41
Tumor location (colon/rectum)	3	NA	2.19 [1.74, 2.77]; P=0.00^*^	I^2^ = 0.04%; P=0.37

NA, not apply; HIV, human immunodeficiency virus.

### 3.4 OS of the patients

**Figure 2:**
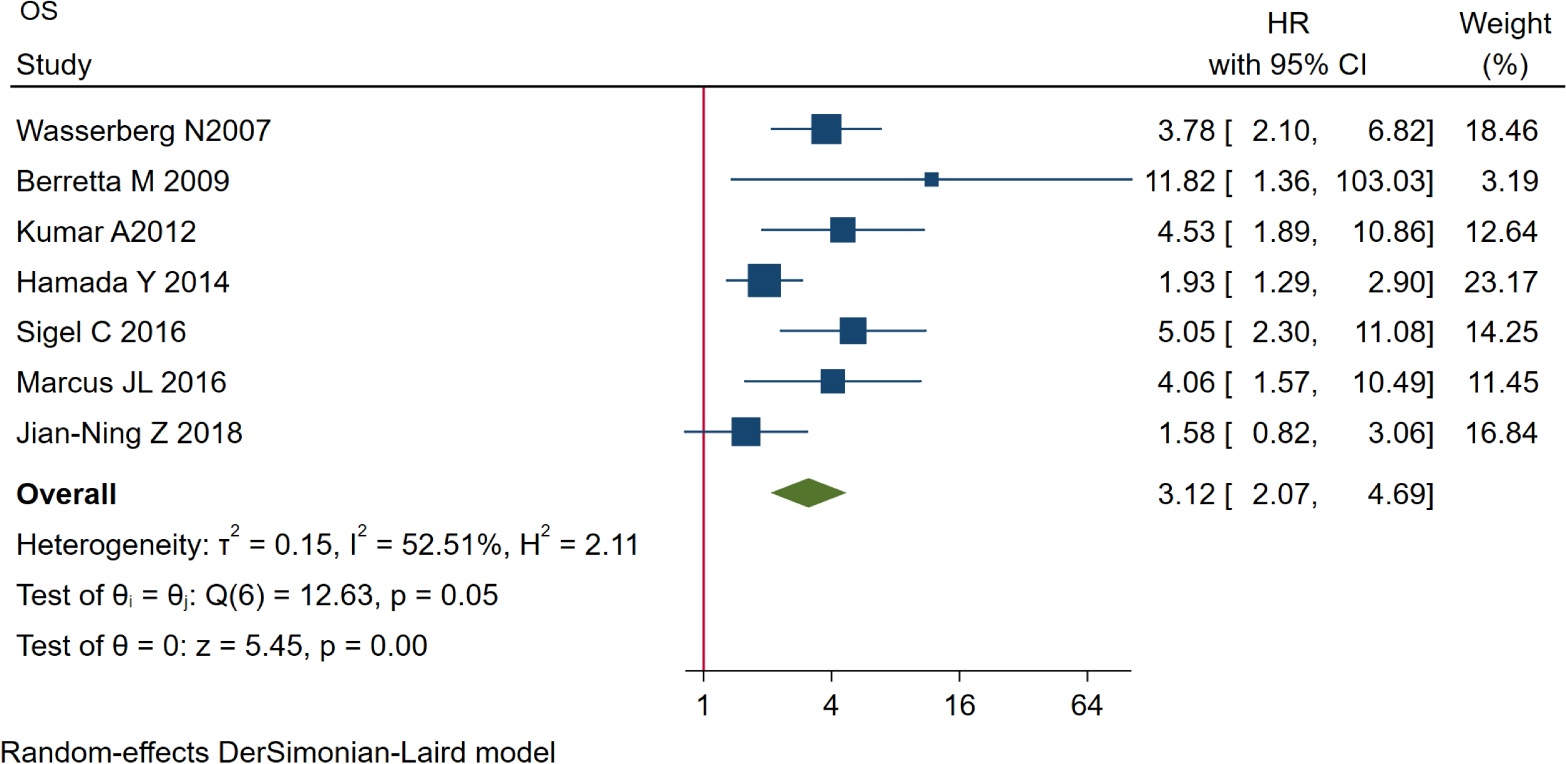
Comparison of OS between the two groups. OS, overall survival.

According to our data analysis, we found that HIV was associated with worse OS in CRC patients (HR=3.12, 95% CI=2.07 to 4.69, I^2^ = 52.51%, P=0.00<0.1) (**Figure 2**).

### 3.5 Funnel plot

**Figure 3:**
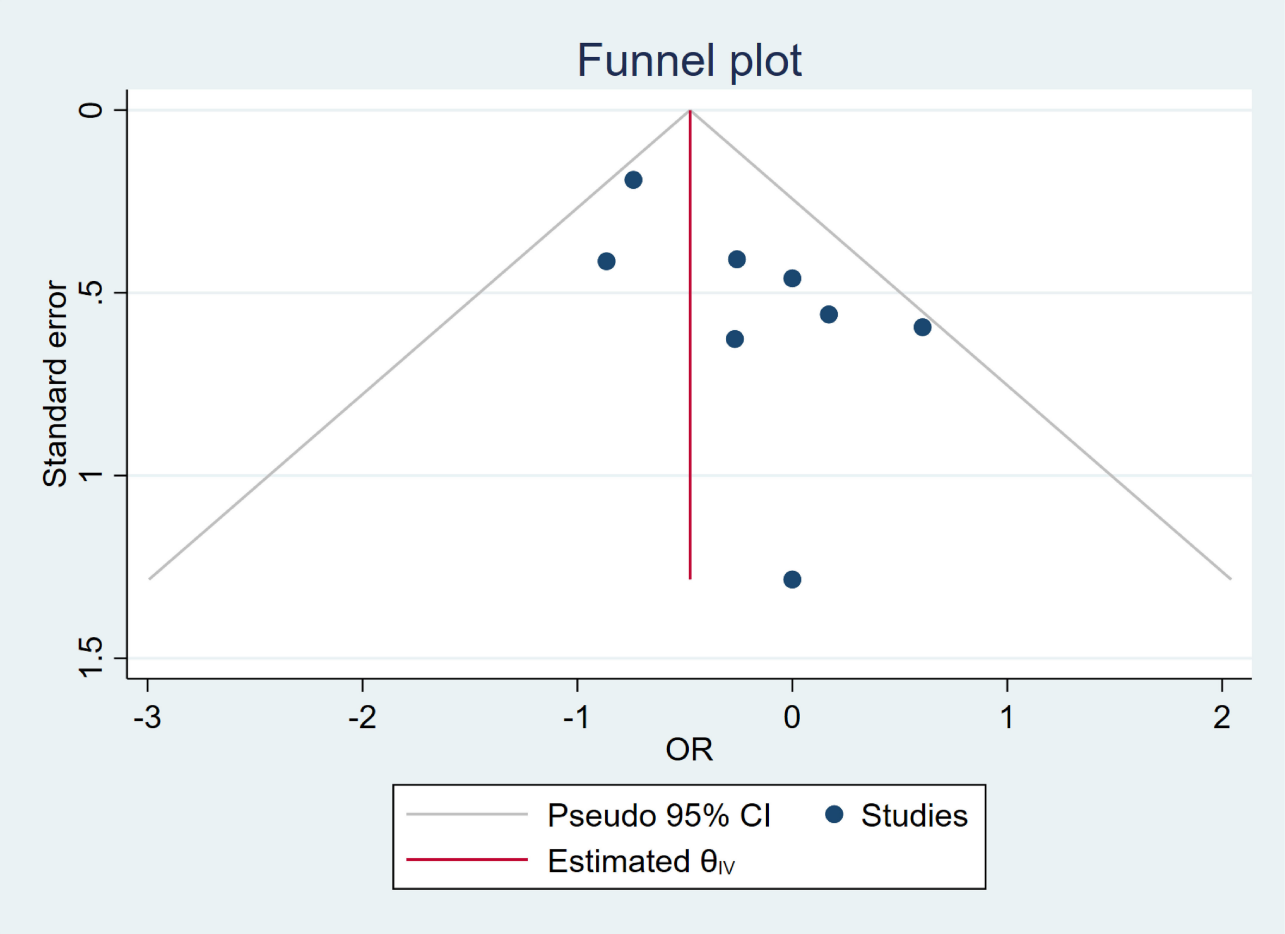
Funnel plot of overall complications.

Funnel plot was used to assess publication bias. According to the funnel plot, which showed symmetry, suggesting that there was no significant publication bias in the included studies. Symmetry in the plot typically indicated that the studies were evenly distributed around the effect estimate, supporting the reliability and generalizability of the results (**Figure 3**).

### 3.6 Sensitivity analysis

**Figure 4:**
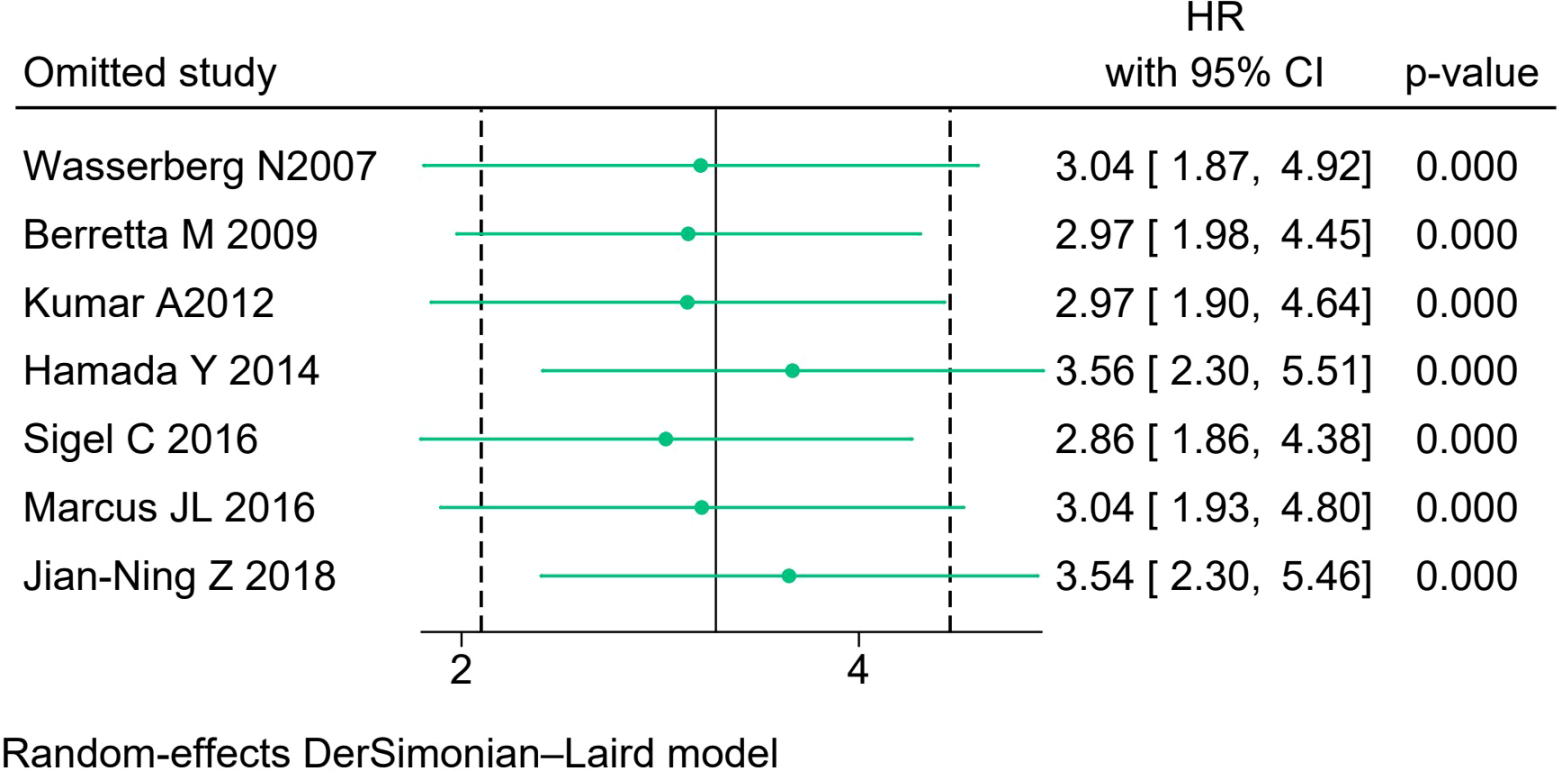
Sensitivity analysis.

We analyzed sensitivity by excluding each study one at a time. There were no significant differences in the results after each analysis was performed (**Figure 4**).

## 4 Discussion

A total of eight studies including 2180 patients were enrolled in this current study. We pooled up baseline information and prognosis of the included patients. After pooling up the data, we found the HIV group had less males, a higher tumor grade and a higher proportion of colon cancer. Moreover, the HIV group had a worse OS than the non-HIV group, however, the heterogeneity was not low and which needed to be considered.

The survival of HIV-infected patients was prolonged in the HAART era, and many HIV-positive CRC patients required radical surgery (27–30). Clinical practice shown that surgical trauma or stress response could affect the immune function of the patient’s body, stimulate the immune system in mild cases and strengthen the immunity of the body, but produce immunosuppression in severe cases and trigger the development of opportunistic infections such as bacteria, tuberculosis, fungi, and viruses. The higher HIV-RNA, the higher infectiousness, and the lower CD4+ count, the higher the risk of opportunistic infection (31). This could increase the incidence of postoperative complications and morbidity and mortality to some extent.

The researchers focused on finding out the impact of HIV on CRC patients after surgery. However, there was still a controversy. Some studies did not find a significant impact of HIV on OS in CRC patients (17–21). In their respective studies, Berretta M and Sigel C et al. showed that patients with HIV-infected CRC had a worse OS after surgery than the general CRC patients (15, 16). Previous studies have reported the survival in HIV-positive CRC patients, however, this meta-analysis is the first to specifically focus on CRC.

In this current study, the outcome revealed that HIV-infected patients had a worse OS in CRC patients after surgery, however, the heterogeneity of the results was not low and needs to be taken into consideration. Similarly, previous studies found a reduction in OS in HIV-associated cancers, such as malignant melanoma, other skin cancers, head and neck tumors, and lung cancer (32–35). The mechanisms of HIV altered clinical course of these malignancies were unclear. In acquired immunodeficiency syndrome (AIDS)-associated cancers (so-called AIDS-defining malignancies), most of the pathogenesis was virus-related, with impaired viral immunity being the cause of persistent viral pathogens. In the case of non-AIDS-defining malignancies, aggressive tumor behavior might be associated with an altered host immune response in immunosuppressed patients. Two epidemiological studies shown an association between advanced immunosuppression associated with HIV disease progression and non-AIDS-defining malignancies (36, 37).

Moreover, a study found low CD4 counts were associated with early recurrence in HIV-positive malignant melanoma patients (38). In contrast, when comparing AIDS to cancer registries, Mbretier et al. did not find an association between CD4 counts and non-AIDS defining cancers in over 82,000 patients (39). CD4 T cells were central to antitumor immunity, as they regulate immune surveillance and coordinate the cytotoxic response against malignant cells. Chronic immune suppression, as seen in HIV-positive individuals, could potentially compromise these mechanisms, leading to worse outcomes in certain cancers. At present, the current study found no correlation between CD4 counts and CRC occurrence, as all patients had relatively high CD4 counts at the time of CRC diagnosis (17). This finding may reflect effective antiretroviral therapy (ART) in maintaining immune function, which could mitigate the impact of CD4 count depletion. However, it is worth exploring whether variations in CD4 counts, particularly during different stages of HIV infection or treatment, might influence CRC prognosis or response to therapy. Future research could focus on longitudinal studies to better elucidate the dynamic interplay between immune status and CRC outcomes in HIV-positive populations.

We also found that the HIV group had more colon cancer. This was consistent with previous study (40). The HIV group had a higher tumor grade than the non-HIV group. High tumor grade might mean high aggressiveness and greater potential for metastasis. This could also be one of the reasons why the HIV group had a worse OS, but more studies were needed to prove this.

To our best knowledge, this study was the first study to pool up the prognosis in HIV-infected CRC patients in previous studies. However, there were some limitations of this pooling up analysis. First, most of these studies focused on Europe, which might cause selection bias; Second, insufficiency of the patient’s baseline information; Third, missing other factors on OS; Fourth, the results was moderate heterogeneity with no clear source of heterogeneity, however, there was insufficient data to perform a subgroup analysis. Therefore, more and more detailed research on this is needed in the future.

## 5 Conclusion

Patients with HIV-infected CRC have a worse prognosis than the general CRC patient. As people living with HIV live longer, these patients should also receive more attention.

## Data Availability

The raw data supporting the conclusions of this article will be made available by the authors, without undue reservation.

## Supplementary material

The Supplementary Material for this article can be found onlineat: https://www.frontiersin.org/articles/10.3389/fonc.2025.1440105/full#supplementary-material
