# Supplementary material for: Patients with colorectal cancer combined with HIV had a worse overall survival after surgery: a meta-analysis
Source: Front Oncol. 2025 Jan 29;15:1440105. doi: 10.3389/fonc.2025.1440105 (PMC11813759; doi:10.3389/fonc.2025.1440105)
Supplement: Supplementary file 1 [file Table1.docx]

Table S1 Results of quality assessment using the Newcastle-Ottawa Scale for case- control studies.

| Study | Selection |  |  |  | Comparability | Exposure |  |  | Scores |
| --- | --- | --- | --- | --- | --- | --- | --- | --- | --- |
|  | Adequate definition of cases | Representati-veness of the cases | Selection of controls | Definition of controls | Control for important factor^#^ | Ascertainment of exposure | Same method of ascertainment for cases and controls | Non-response rate |  |
| Wasserberg N, 2007 | ⭐ | ⭐ | ⭐ | ⭐ | ⭐⭐ | ⭐ | ⭐ | ⭐ | 9 |
| Berretta M, 2009 | ⭐ | ⭐ | ⭐ | ⭐ | ⭐⭐ | ⭐ | ⭐ | ⭐ | 9 |
| Kumar A, 2012 | ⭐ | ⭐ | ⭐ | ⭐ | ⭐ | ⭐ | ⭐ | ⭐ | 8 |
| Hamada Y, 2014 | ⭐ | ⭐ | ⭐ | ⭐ | ⭐⭐ | ⭐ | ⭐ | ⭐ | 9 |
| Sigel C, 2016 |  | ⭐ | ⭐ | ⭐ | ⭐ | ⭐ | ⭐ | ⭐ | 7 |
| Pillay SK, 2022 | ⭐ | ⭐ | ⭐ | ⭐ | ⭐⭐ | ⭐ | ⭐ | ⭐ | 9 |
| Marcus JL, 2016 | ⭐ | ⭐ | ⭐ | ⭐ | ⭐ | ⭐ | ⭐ | ⭐ | 8 |
| Jian-Ning Z, 2018 |  | ⭐ | ⭐ | ⭐ | ⭐ | ⭐ | ⭐ | ⭐ | 7 |

Note: ^#^A maximum of 2 stars can be allotted in this category, one for age, the other for other controlled factors.
